# Involvement of Sphingolipids in Ethanol Neurotoxicity in the Developing Brain

**DOI:** 10.3390/brainsci3020670

**Published:** 2013-04-26

**Authors:** Mariko Saito, Mitsuo Saito

**Affiliations:** 1 Division of Neurochemistry, Nathan S. Kline Institute for Psychiatric Research, 140 Old Orangeburg Rd., Orangeburg, NY 10962, USA; 2 Department of Psychiatry, New York University Langone Medical Center, 550 First Ave., New York, NY 10016, USA; 3 Division of Analytical Psychopharmacology, Nathan S. Kline Institute for Psychiatric Research, 140 Old Orangeburg Rd., Orangeburg, NY 10962, USA; E-Mail: mitsaito@nki.rfmh.org

**Keywords:** ethanol, sphingolipid, developing brain, apoptosis, neurodegeneration, mitochondria, ceramide, ganglioside, sphingosine-1-phosphate, fetal alcohol spectrum disorders

## Abstract

Ethanol-induced neuronal death during a sensitive period of brain development is considered one of the significant causes of fetal alcohol spectrum disorders (FASD). In rodent models, ethanol triggers robust apoptotic neurodegeneration during a period of active synaptogenesis that occurs around the first two postnatal weeks, equivalent to the third trimester in human fetuses. The ethanol-induced apoptosis is mitochondria-dependent, involving Bax and caspase-3 activation. Such apoptotic pathways are often mediated by sphingolipids, a class of bioactive lipids ubiquitously present in eukaryotic cellular membranes. While the central role of lipids in ethanol liver toxicity is well recognized, the involvement of sphingolipids in ethanol neurotoxicity is less explored despite mounting evidence of their importance in neuronal apoptosis. Nevertheless, recent studies indicate that ethanol-induced neuronal apoptosis in animal models of FASD is mediated or regulated by cellular sphingolipids, including via the pro-apoptotic action of ceramide and through the neuroprotective action of GM1 ganglioside. Such sphingolipid involvement in ethanol neurotoxicity in the developing brain may provide unique targets for therapeutic applications against FASD. Here we summarize findings describing the involvement of sphingolipids in ethanol-induced apoptosis and discuss the possibility that the combined action of various sphingolipids in mitochondria may control neuronal cell fate.

## 1. Introduction

Sphingolipids, which are a class of bioactive lipids containing sphingoid bases as a basic structure, are involved in various cellular processes, such as differentiation, proliferation and apoptosis in a wide variety of cellular systems (reviewed by [1,2]). There are numerous derivatives, including sphingosine, sphingosine-1-phosphate, ceramide, ceramide-1-phosphate, sphingomyelin, and glycosphingolipids (reviewed by [3,4]). Apoptosis triggered by various inducers is often mediated or regulated by sphingolipids. Specifically, ceramide and sphingosine have been recognized as pro-apoptotic mediators, while sphingosine-1-phosphate (S1P) has been considered anti-apoptotic (reviewed by [2,5,6,7]). Such involvement of sphingolipids in cell death and survival has been widely observed in the nervous system (reviewed by [8,9,10,11,12]), which is highly enriched in sphingolipids (reviewed by [13]). While ceramides may be necessary to regulate neural cell numbers during brain development [14,15,16,17], dysregulated ceramide formation is involved in neurodegeneration in several neurodegenerative diseases (reviewed by [11,18,19,20]), and certain gangliosides (sialic acid-containing glycosphingolipids), such as GM1, often exert neuroprotection (reviewed by [21,22,23,24,25,26]).

Ethanol affects lipid metabolism in many cell types, and such alterations are considered a factor causing or regulating tissue injury. For example, in the liver, alcoholic steatosis is recognized as a condition leading to steatohepatitis, fibrosis, and cirrhosis (reviewed by [27]). It has been shown that, along with enhanced lipogenesis induced by ethanol metabolism (reviewed by [28]), many regulators of lipid metabolism including AMP-activated protein kinase (AMPK) and sterol regulatory element-binding protein (SREBP)-1 are disturbed by ethanol, causing hepatic steatosis (reviewed by [29]). The disturbance in lipid metabolism by ethanol is also associated with pro-apoptotic ceramide elevation [30], (reviewed by [31]).

Prenatal ethanol exposure perturbs brain development in all three trimesters of pregnancy, leading to long-lasting deficits in cognition and behavior observed in patients with fetal alcohol spectrum disorders (FASD) (reviewed by [32]). To elucidate mechanisms of ethanol toxicity in the developing brain, rodent models of FASD have been widely used (reviewed by [33]). As reviewed by Guerri [34], ethanol exposure in rats during the period between gestational day (GD) 5 and GD11 (roughly equivalent to the first trimester of human gestation) results in neural tube defects and alterations in neural precursor cell proliferation. Ethanol exposure during the period between about GD11 and GD18 (roughly equivalent to the second trimester) alters development of radial glia and disturbs proliferation, generation, and migration of neurons, and ethanol exposure during the period between about GD18 and postnatal days (P) 9 (roughly equivalent to the third trimester) induces severe neuronal loss, reactive gliosis, and delayed myelination.

While sphingolipids are likely to be involved in these diverse effects of prenatal ethanol, the majority of *in vivo* studies so far have focused on testing the involvement of sphingolipids in ethanol-induced neuronal death. Ethanol-induced neurodegeneraion is particularly robust during the period of active synaptogenesis [35,36] (corresponding to the first two postnatal weeks for rodent pups and the third trimester for human fetuses), and likely contributes to the pathogenesis and outcome of FASD [35,37,38] (reviewed by [32]). This neuronal death occurs via the Bax-dependent mitochondria-mediated apoptotic pathway [39,40,41,42], and may be related to ethanol-induced changes in lipid metabolism. In fact, ethanol profoundly alters lipid metabolism in the developing brain and cultured neurons. Studies show it induces ceramide elevation [43,44,45], alters fatty acid composition [46,47], changes ganglioside profiles [45,48,49], and promotes ceramide/sphingosine recycling for ganglioside biosynthesis [50]. These effects of ethanol on sphingolipid metabolism and accumulated evidence on the roles of ceramides in mitochondria-mediated neuronal apoptosis strongly suggest that ethanol-induced apoptotic neurodegeneration is mediated or regulated by altered sphingolipid metabolism. In this review, we summarize studies related to this hypothesis, focusing on roles of ceramides, S1P, and gangliosides in ethanol-induced apoptosis in the developing brain, and discuss the possible functions of these sphingolipids in mitochondria. We also present the possibility that the developing brain at the peak of active synaptogenesis may display unique sphingolipid profiles/metabolism, which contribute to its heightened sensitivity to the apoptotic effects of ethanol. First, we briefly summarize studies showing mechanisms behind ethanol-induced apoptosis in the developing brain. Then, we describe the possible involvement of ceramide, S1P, and gangliosides in this apoptotic pathway.

## 2. Ethanol-Induced Neuronal Apoptosis in the Developing Brain

### 2.1. Neuronal Apoptosis Triggered by Ethanol during the Period of Synaptogenesis

Previous studies using rodent models for FASD have demonstrated that early postnatal binge ethanol exposure during the brain growth spurt period, on postnatal days 4–9 (P4–9) for example, causes high incidence of neuronal loss [51,52,53,54], followed by long-lasting behavioral deficits [52,55,56,57]. The cell loss appears to be caused by apoptosis, which occurs immediately after ethanol exposure, and is detected by morphological characteristics of apoptosis (such as cell shrinkage), TUNEL staining, caspase-3 activation, and involvement of the Bcl-2 family [35,36,58,59,60]. The developing neurons are sensitive to the pro-apoptotic effects of ethanol during the brain growth spurt period, which is also the period of active synaptogenesis, although different neuronal populations display the peak sensitivity at different time points within this window [35]. Acute ethanol exposure in P7 rodents induces apoptotic neurodegeneration within one day in many brain regions, including the cortex, thalamus, and caudate putamen [35,36] in a dose-dependent manner [61], and results in long-lasting behavioral [38,62,63,64,65] and electrophysiological [38,64] deficits observed in adult animals. Acute ethanol also triggers apoptosis of Purkinje and granule cells in the cerebellum of neonatal rodents, and the peak apoptosis is found around P4 [40,41,66,67,68,69,70], (reviewed by [71]). Such acute ethanol-induced apoptotic neurodegeneration during the brain growth spurt is observed not only in rodents but also in fetal macaque brain at various stages of gestation (G105 to G155) depending on the brain regions [37]. Chronic ethanol exposure during gestation and lactation also induces apoptosis in the early postnatal rat brain, although cell death is found more in GFAP-positive glial cells [72]. The effects of ethanol on astrocytes appear to depend on the levels of ethanol, duration, timing of exposure, and the stage of glial maturation [73] as shown in the effects of ethanol on neurons. While prenatal ethanol exposure reduces GFAP expression [73], brief exposure to high levels of ethanol during the brain growth spurt causes astrogliosis detected by an increase in immunoreactive GFAP [74], along with microglial activation that appears to facilitate clearance of dead neurons [75]. Ethanol-induced apoptosis is reported in many types of cultured neurons as well [76,77,78,79,80], (reviewed by [71]). Thus, developing neurons are particularly sensitive to the pro-apoptotic effects of ethanol during the period of synaptogenesis. 

### 2.2. Mechanisms behind Ethanol-Induced Neuronal Apoptosis

Ethanol exposure in P7 rats or mice induces apoptosis via mitochondria-mediated intrinsic pathway, involving Bax-induced disruption of mitochondrial membranes, cytochrome c release, and caspase-3 activation in the neonatal brain [39,42]. Although direct targets of ethanol leading to the intrinsic apoptotic pathway have not been fully elucidated, ethanol’s blocking action at NMDA receptors and its enhancing action at GABA_A_ receptors may be responsible [35,36]. Ethanol exposure in P4–6 rats decreases Purkinje cell expression of TrkB and TrkC receptors [81], and apoptotic neurodegeneration in P7 mice exposed to ethanol is associated with inactivation of Akt [82,83,84] and extracellular signal-regulated kinase (ERK) [83,84] and activation of glycogen synthase kinase-β (GSK3β) [75,82]. Lithium, a GSK3β inhibitor [85], attenuates ethanol-induced apoptosis in the P7 brain [82,84,86], (reviewed by [87]). It is also reported that ethanol-induced apoptosis is accompanied by c-Jun *N*-terminal kinase (JNK) activation in neonatal rats [39,88]. Ethanol-induced oxidative stress/free radical formation is one of the important factors linked to the apoptotic pathway as well. Ethanol rapidly increases reactive oxygen species (ROS) and the lipid peroxidation product, 4-hydroxynonenal (HNE), in the neonatal brain [89,90]. Importantly, various anti-oxidant treatment paradigms ameliorate cell death triggered by ethanol [90,91,92]. NADPH oxidase (NOX) activation by ethanol in the P7 mouse brain seems to be a cause of ROS generation [93]. Such oxidative stress induced by ethanol may trigger the endoplasmic reticulum (ER) stress reported in the P7 brain [94].

Also, in cultured neurons, ethanol induces apoptosis via mitochondria-mediated intrinsic pathway, involving Bax-induced disruption of mitochondrial membranes, cytochrome c release, and caspase-3 activation [41,80,95,96]. Ethanol may trigger apoptosis in cultured rat cerebellar granule neurons (CGNs) by inhibiting NMDA receptor functions [97] or inhibiting insulin-like growth factor-1 (IGF-1) receptor functions [98]. The inhibition of NMDA receptors leads to the suppression of brain-derived neurotrophic factor (BDNF) expression [99,100], and the inhibition of BDNF or IGF-1 function appears to induce apoptosis through the inhibition of pro-survival PI3K/Akt [101] in CGNs [98,99] and in cortical neurons [102]. The inhibition of PI3K/Akt pathway triggers caspase-9 and caspase-3 activation, which is an execution phase of apoptosis [103]. The importance of GSK3β activation in ethanol-induced neuroapoptosis has been also highlighted by studies *in vitro*; overexpression of GSK3β sensitizes neurons to ethanol toxicity [104], and lithium attenuates ethanol-induced apoptosis in cultured neurons [86]. Furthermore, SB216763, a selective GSK3β inhibitor, prevents apoptosis induced by ethanol in cultured neurons [105]. It is also reported that ethanol-induced apoptosis is accompanied by JNK activation in SK-N-SH cells [106]. In glial cell cultures, cell death triggered by ethanol is associated with activation of JNK [107,108], mitogen-activated protein kinase p38 (p38 MAPK) [107,108], and ERK pathways [107,108,109]. Also, ethanol rapidly increases ROS and 4-HNE, in cultured neurons [95,110,111,112,113,114], and various anti-oxidant treatment paradigms ameliorate cell death triggered by ethanol [95,110,112,113,115]. Further, the neuronal glutathione content appears to determine selective vulnerability of cultured cortical neurons to ethanol-induced apoptosis [116]. It is indicated that metabolism of ethanol generates ROS and nitric oxide (NO) by activation of NADPH oxidase (NOX)/xanthine oxidase and inducible NO synthase in cultured cortical neurons [111]. 

Thus, ethanol activates a mitochondria-mediated, Bax-dependent apoptotic pathway, involving ROS formation, inactivation of Akt and ERK, and activation of GSK3β and JNK. Accumulated evidence suggests that sphingolipids play important roles in such apoptotic pathways. Specifically, ceramide has been recognized as a pro-apoptotic mediator, while sphingosine-1-phosphate (S1P) and GM1 ganglioside have been considered anti-apoptotic mediators (reviewed by [2,6,7,21,22,23,26]). The relevance of ceramide, S1P, and gangliosides in ethanol-induced apoptosis is described in the following sections. 

## 3. Ceramide Involvement in Ethanol-Induced Neuronal Apoptosis in the Developing Brain

### 3.1. Involvement of Ceramide in Apoptosis in the Developing Brain

**Figure 1:**
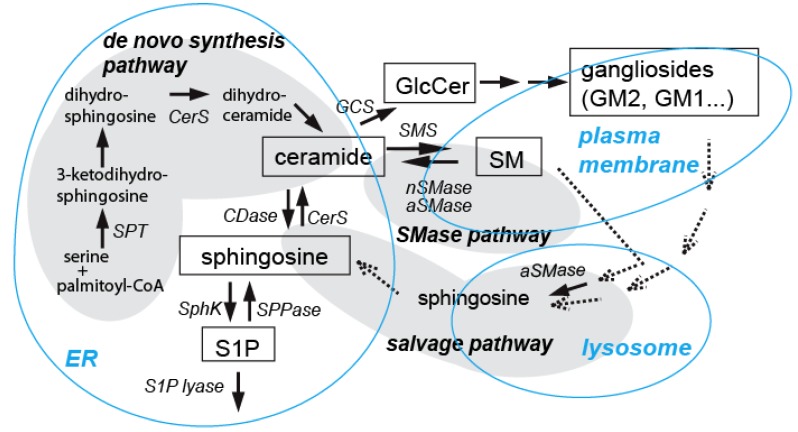
Ceramide generating pathways. Three major pathways for ceramide generation are shown here. Ceramide is synthesized via “*de novo synthesis pathway*” in endoplasmic reticulum (ER), which involves several enzymes including serine palmitoyltransferase (SPT, the initial sphingolipid synthesizing enzyme) and ceramide synthase (CerS). Ceramide can be generated by activation of neutral (n) or acid (a) SMases (“*SMase pathway*”), often found in the plasma membrane. In the “*salvage pathway*”, ceramide is synthesized by CerS from sphingosine released from the lysosome. Although not shown here, ceramide generation may also occur in the mitochondria, where ceramide generating enzymes, such as CerS, have been found. These pathways are activated by a variety of apoptotic inducers in various cell types including neurons as described in the text. (CDase, ceramidase; SphK, sphingosine kinase; SPPase, S1P phosphatase; GCS, glucosylceramide synthase; SMS, sphingomyelin synthase; GlcCer, glucosylceramide.)

Ceramides are essential sphingolipid messengers regulating a diverse range of cell-stress responses, such as apoptosis, cell senescence, and autophagy. Various factors, including the species of ceramides generated (out of >200 structurally distinct molecules) and its subcellular localization, appear to determine the ceramide functions (reviewed by [117]). Numerous studies have demonstrated that ceramide mediates or enhances both extrinsic and intrinsic apoptotic pathways in many cell types (reviewed by [1,2,3,118]) including neurons (reviewed by [8,9,10,11,12,119,120]). While such ceramide-mediated apoptosis can be beneficial during a certain period of neuronal development for regulating neural cell numbers [14,15,17,121], dysregulated ceramide formation is implicated in neural death in several neuroinflammatory and neurodegenerative disorders (reviewed by [8,11,12,18,19,20]). A variety of studies using cultured neurons and animal models of neurodegenerative diseases support the notion that ceramide is involved in the apoptotic pathways (reviewed by [8,9,10,11,12,119,120,122]). First, cellular ceramide elevation, either by adding natural or short-acyl chain analogs of ceramide or by modulating ceramide metabolizing enzymes, induces apoptosis in cultured neurons (reviewed by [11,119,122]). Second, many apoptotic inducers elevate endogenous levels of ceramide, and the inhibition of such ceramide generation by pharmacological or genetic manipulation attenuates cell death (reviewed by [8,11,12,20,119,120]). As shown in Figure 1, ceramide can be generated by activation of neutral or acid sphingomyelinase (SMase), by activation of the salvage pathway, which involves ceramide formation from sphingosine released from the lysosome, or by enhancement of *de novo* ceramide synthesis (reviewed by [2,4,11,20,120]). In general, neutral and acidic SMases trigger early and transient ceramide increase, while *de novo* ceramide synthesis causes late and sustained ceramide elevation [123]. Recent studies indicate that molecular species of ceramides thus produced is an important factor in determining ceramide functions [117]. In untreated cultured neurons, C18 is a major fatty acid of ceramides [124], and ceramide synthase 1 (CerS1) that catalyzes *de novo* synthesis of C18 ceramide is a major and specific CerS in neurons [125] out of six mammalian CerSs (CerS1–CerS6) (reviewed by [126]). However, the increase in C16 ceramide is associated with apoptosis in neurons [124,127,128] as well as in some other cell types [129,130,131], while increases in C20 and C24 ceramides in hippocampal tissues from an Alzheimer’s disease (AD) mouse model are linked to astroglial cell death [132]. 

The ceramide elevation in the brain or in cultured neurons is followed by activation of pro-apoptotic signaling pathways, p38 MAPK [133,134], JNK [134,135], and GSK3β [135]. In concert with activation of these pro-apoptotic pathways, ceramide elevation is associated with inhibition of survival pathways, PI3K/Akt [135,136] and ERK pathways [133,135]. The inactivation of these pathways may be caused by direct activation of protein phosphatase 2A (PP2A) by ceramide [137], followed by dephosphorylation (inactivation) of Akt and ERK ([138], reviewed by [139,140]).

Ceramide is also implicated in mediating oxidative stress via direct effects on mitochondrial ROS generation or via activation of NOX (reviewed by [141,142]). For example, studies show that ceramide accumulation by TNFα-stimulated neutral SMase activation results in the formation of ROS by NOX activation in dorsal root ganglion neurons [143]. 

Thus, while ceramide is involved in many apoptotic pathways, mechanisms of ceramide formation, sites of the ceramide action, ceramide species generated, and the downstream pathways may differ depending on the type of apoptotic inducers and cellular conditions. However, it is generally recognized that mitochondria are key sites of the ceramide action in a variety of apoptotic pathways [144]. Specifically, the role of mitochondrial ceramide (or ceramide metabolites) in Bax/Bak-dependent mitochondrial outer-membrane permeabilization (MOMP) and the following release of mitochondrial proteins (including cytochrome *c*) have been reported not only in mammalian cells [144,145,146,147,148], but also in yeast cells [146,149]. The brain mitochondria contain SM and ceramide [150], and the related enzymes, such as ceramide synthases (CerS1, CerS2, CerS4 and CerS6) [15,128], SMases [151], and neutral ceramidase [152], providing potentially important intracellular compartment for ceramide metabolism [153]. The elevation of ceramide content and CerS activity in mitochondrial ceramide-rich microdomain appears to be necessary for Bax insertion, MOMP, and the following release of pro-apoptotic factors in radiation-induced apoptosis [148]. Ceramides in mitochondria are also implicated in regulation of Ca^2+^ levels. Studies indicate that ceramide is responsible for increased Ca^2+^ levels in mitochondria prior to calpain-mediated apoptosis in retinal photoreceptor cells [154] and in primary oligodendrocyte precursors [15]. CerS6/ceramide in mitochondrial inner membrane is suggested to regulate mitochondrial Ca^2+^ homeostasis by inhibiting mitochondrial permeability transition pore (MPTP) opening [15]. Because excessive accumulation of Ca^2+^ in mitochondrial matrix can trigger MPTP opening at a high conductance state and lead to cell death by necrosis, MPTP may regulate necrosis [153] in contrast to MOMP, which releases small pro-apoptotic molecules, leading to apoptosis. It is indicated that MPTP-dependent, apoptosis-independent process is critical for brain injury in the adult, whereas Bax-dependent apoptotic mechanisms prevail in the immature brain [155]. This notion may be related to studies indicating that mitochondrial Ca^2+^-loading capacity and the threshold of MPTP opening are higher in immature brain [153], probably due to the elevated expression of C16-ceramide (and CerS6) in mitochondria [15]. These studies suggest that ceramides regulate apoptosis by affecting both MOMP and MPTP, although further investigation is necessary to reveal precise roles of mitochondrial ceramides in neuronal apoptosis in the developing brain.

### 3.2. Ceramide in Ethanol-Induced Apoptosis in the Developing Brain

As described above, ethanol-induced apoptosis in the developing brain shows similar characteristics to those observed in the ceramide-mediated apoptotic pathway. Both ethanol and ceramide activate mitochondria-mediated apoptotic pathways involving Bax-induced disruption of mitochondrial membranes, ROS formation, inactivation of Akt and ERK, and activation of GSK3β and JNK, suggesting that ceramide is involved in ethanol-induced apoptosis. 

In cultured neurons, our studies [45] have shown that ethanol-induced cell death in CGNs and SK-N-SH human neuroblastoma cells are associated with significant accumulation of ceramide. Further, ethanol-induced cell death in these neurons is attenuated by myriocin, an inhibitor of serine palmitoyltransferase (SPT) (the first rate limiting enzyme for sphingolipid synthesis, as shown in Figure 1), implying that *de novo* ceramide synthesis is important for this ethanol-induced cell death. Concomitant increases in levels of triglycerides (TG) and GM2 ganglioside [45] indicate that ethanol may enhance lipid synthesis or inhibit fatty acid β-oxidation, leading to ceramide accumulation, as reported in the liver [30], although our studies have also shown that glucosylceramide (GlcCer) decreases by ethanol treatment in these neurons. Given that GlcCer synthase expression protects against ceramide-induced stress in keratinocytes [156] and elevation of GlcCer is detected in multidrug-resistant cancer cells [157], GlcCer itself or the ceramide/GlcCer ratio may be important in apoptotic/survival pathways. 

**Figure 2:**
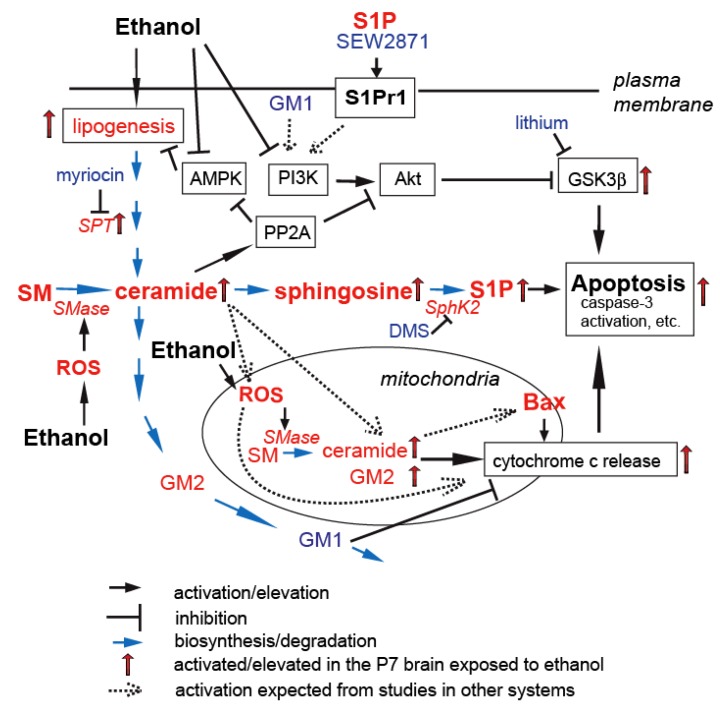
Sphingolipid involvement in ethanol-induced apoptosis in the P7 mouse brain. This figure, which summarizes possible involvement of ceramide, sphingosine-1-phosphate (S1P), GM2 and GM1 in ethanol-induced apoptosis, is based on previous studies by us and others. Ceramide generated via enhanced *de novo* synthesis and SM hydrolysis inactivates PI3K/Akt pathway and activates GSK3β. Ceramide may also directly affect mitochondrial membrane permeability. Increases in GM2 and S1P may enhance apoptosis while exogenous GM1 shows neuroprotection. ROS generation and AMPK inhibition triggered by ethanol are likely to be linked to the altered sphingolipid metabolism during apoptosis. The coordinate action of sphingolipids in mitochondria may be crucial for the regulation of the ethanol-induced, mitochondria-mediated apoptotic pathway.

Increases in ceramide, TG, and GM2 are also observed *in vivo* in the brain 4 to 24 h after P7 mice are acutely exposed to ethanol (2.5 g/kg, s.c., twice with a 2 h interval as described in [36]) [43,44,49]. Ceramide elevation is specifically prominent in brain regions where strong neurodegeneration occurs [43]. Such ceramide elevation as well as apoptotic neurodegeneration assessed by cleaved (activated) caspase-3 immunostaining and Fluoro-Jade staining is attenuated by SPT inhibitors (myriocin and l-cycloserine) [43]. Concomitantly, SPT immunostaining is enhanced in cleaved caspase-3 positive neurons, and the SPT activity increases in ethanol-treated forebrain samples [43]. These results suggest the importance of *de novo* ceramide synthesis in ethanol-induced apoptosis, which agrees with our studies using cultured neurons [45]. However, the contribution of SMase activation by ethanol cannot be excluded, because small but significant increases in both neutral and acid-SMase activity are observed when forebrain slices from P7 mice are treated with ethanol *in situ* [158]. The neuronal localization of SPT [43] and our preliminary data indicating strong ceramide staining in cleaved caspase-3 positive neurons [159] suggest that increased ceramide is localized mainly in neurons. The inhibition of AMPK and activation of acetyl-CoA carboxylase (a lipogenic enzyme) found in the P7 brain exposed to ethanol [44] suggest that ethanol-enhanced lipogenesis may be linked to the ceramide elevation as indicated in other organs [160,161]. However, it has been also proposed in the liver that the effects of ethanol on AMPK inactivation (dephosphorylation) may be mediated by the direct activation of protein phosphatase 2A (PP2A) by ceramide [137] produced by activated acid SMase [162,163]. It is possible that ethanol induces both transient SMase activation and long-lasting enhancement of *de novo* ceramide synthesis. Figure 2 illustrates the possible involvement of ceramide in the ethanol-induced apoptotic pathway in the P7 mouse brain. Because apoptotic neurons produced by P7 ethanol exposure are cleared promptly by activated microglia, which return to the shape of resting microglia within 48 h [75], it is expected that lipid alterations, including ceramide elevation, are short-lived. However, neonatal ethanol exposure has been shown to induce persistent neocortical astrogliosis [164,165] and increased cytokine (such as TNF-α) formation [165] in adolescent rats, suggesting that neonatal ethanol exposure induces prolonged neuroinflammation. It has been reported that administration of ethanol to pregnant mice on GD15–16 induces elevation of ceramide and sphingosine in the brain of juvenile progeny mice [166]. Whether the elevation of these lipids in the juvenile brain is associated with cell death and/or neuroinflammation, or whether P7 ethanol exposure induces long-term ceramide elevation remains to be explored. 

Ceramide has been also linked to ethanol-induced apoptosis in neural crest-derived cells both *in vivo* and *in vitro* [167]. In this case, ceramide appears to increase by enhanced SM hydrolysis or impaired conversion of ceramide to SM [167]. In cultured astrocytes, ethanol-induced cell death is associated with ceramide elevation via activation of neutral and acid SMases, along with activation of JNK, p38, and ERK [107]. Ethanol-induced oxidative stress may activate SMases because changes in intracellular redox seem to regulate neutral SMases (reviewed by [141]). It is also suggested that ethanol induces astroglial apoptosis by disrupting phospholipase D signaling, thereby reducing phosphatidic acid and increasing ceramide formation [168]. 

Ceramide, thus elevated by ethanol treatment, may function in mitochondria as suggested in other ceramide-mediated apoptotic pathways. Our preliminary studies [169] indicate that ceramide increases in the mitochondrial fraction isolated from the P7 mouse brain exposed to ethanol, but not in the synaptic plasma membrane or microsomal fractions, where ceramides are mainly localized. A recent study [170] indicates that the interaction of Bax with proteins associated with MPTP is crucial for the initiation/progression of apoptotic cascade in the cerebellum of P4 rats exposed to ethanol. As described in the previous section, ceramide in mitochondria is implicated in the regulation of apoptosis by affecting functions of MOMP and MPTP in several apoptotic models [145,146,147,148,149,153]. Whether ceramide elevated in mitochondria during ethanol-induced apoptosis influences the functions of MOMP and MPTP remains to be explored. 

Thus, apoptosis in the P7 brain exposed to ethanol is associated with changes in lipid metabolism including ceramide elevation. In contrast, ethanol exposure in the P19 mouse brain under the same condition barely induces lipid changes [44] or apoptosis [35,44]. Causes of this heightened sensitivity of the early postnatal rodent brain have not been fully elucidated. However, several factors, which may collectively confer this sensitivity, have been proposed. First, blockade of NMDA receptors induces neuronal death during an early postnatal period [35,171,172,173,174], indicating an important neurotrophic role of NMDA receptor activation during this period in controlling natural programmed cell death (reviewed by [175]) as well as apoptosis induced by ethanol, which is an inhibitor of NMDA receptor functions (reviewed by [176,177]). Secondly, because of the need of the natural programmed cell death, early postnatal neurons are primed to undergo apoptosis [175] by expressing higher levels of apoptotic effectors, such as caspase-3, APAF-1, and Bax [178,179]. Studies show that ethanol exposure in P7 rat brains induces changes in neurotrophic factors, apoptosis-related proteins, antioxidant enzymes, and ROS, which favor both cell death and survival, while ethanol exposure in P21 brains elicits changes, which mostly promote cell survival [59]. In addition, basal levels of nerve growth factor and BDNF are higher in rat brains at P21 than at P7 [59]. It should be also noted that lipid metabolism characteristic to the neonatal brain may confer the additional sensitivity to ethanol-induced apoptosis in these brains. For example, higher expression of CerS6 in neonatal brain [15] may be correlated with the susceptibility of these brains to ethanol-induced apoptosis, because C16 ceramide (a product of CerS6) accumulated in mitochondria inhibits necrotic cell death and promotes apoptosis [15]. Our previous studies indicate that S1P, which is generally neuroprotective, increases during brain development [180]. However, ceramide, TG, lipogenic enzymes (fatty acid synthase and acetyl-CoA carboxylase), and putative lipid metabolism regulators (SREBP-1 and AMPK) are highly expressed in neurons at the early postnatal period and decline thereafter [181]. These results suggest that lipogenesis is more active and ceramide is more readily synthesized in the neonatal brain than in the mature brain. It has been indicated that ceramide produced by chronic ethanol treatment in adult liver is transferred across the blood–brain barrier and causes neurodegeneration [182]. However, during the early postnatal period, ceramide is likely to be synthesized in the brain, although contribution of ceramide generated in the liver cannot be excluded. 

Thus, the involvement of ceramide in ethanol-induced apoptosis is indicated in the neonatal brain. Likewise, elevated ceramide has been linked to apoptosis of neural cells in neurodegenerative diseases and neurological injuries, such as Alzheimer’s disease, HIV-associated dementia, multiple sclerosis, and ischemia/reperfusion injury [8,11,12,18,19,20]. Although mechanisms of ceramide elevation are different among these disorders, ceramide-related activation of mitochondrial cell death pathway as well as JNK activation has been often observed [8,11,12,18,19,20]. However, neuronal cell death processes in these disorders appear to be more complicated than those in ethanol-induced neuronal death in the neonatal brain, because in many cases apoptosis is not the major cell death mode, and the impact of neuroinflammation on cell death is more significant in these disorders [8,11,12,18,19,20]. Ethanol-induced apoptosis in the neonatal brain provides a suitable model to study the mechanisms of sphingolipid involvement in neurodegeneration. Further studies, such as cellular and subcellular localization of increased ceramides, identification of molecular species of these ceramides, and the effects of ethanol on mice and neurons expressing genetically modified enzymes related to ceramide metabolism may offer better understanding of roles of ceramide in ethanol neurotoxicity. Furthermore, ceramide metabolites, such as S1P and gangliosides, may also be involved in ethanol-induced apoptosis, because these lipids are often recognized as apoptosis regulators, and are known to modify ceramide functions as well (reviewed by [7,21,22,23,26,183,184,185]). 

## 4. S1P in Ethanol-Induced Apoptosis in the Developing Brain

It is generally postulated that ceramide induces apoptosis (reviewed by [118]) while S1P promotes cell survival (reviewed by [7]), and the balance between these bioactive lipids, termed “sphingolipid rheostat”, determines the cell fate [7]. This sphingolipid rheostat is mainly regulated by two isoforms of sphingosine kinases, sphingosine kinase 1 (SphK1) and sphingosine kinase 2 (SphK2), which phosphorylate sphingosine to form S1P. The pro-survival effects of S1P produced by SphK1 are usually mediated by the interaction of S1P with five G-protein-coupled cell surface receptors termed S1P receptor 1–5 (reviewed by [7,186]), and the S1P binding to S1P receptors is associated with activation of pro-survival ERK [187], PI3K/Akt [187,188,189,190], and BclXL [189]. However, the pro-apoptotic action of S1P has been reported occasionally [191,192,193,194,195]. Particularly, S1P produced by SphK2 in the nucleus [191] or ER [192,194] is considered pro-apoptotic. 

In the nervous system, S1P plays critical roles in neurogenesis, neurite formation, neuroprotection ([196,197], reviewed by [183]), astrocyte proliferation [198,199,200,201,202], and microglial activation [203]. Most of the SphK activity in the brain appears to be attributed to that of SphK2, which is mainly localized in neurons, while SphK1 is localized primarily in astrocytes [180,204]. It is generally assumed that SphK1 activation exerts a pro-survival influence [205,206], whereas SphK2 activation enhances apoptosis [191]. In fact, S1P produced by SphK2 has been implicated in causing apoptosis through intracellular targets in CGNs derived from S1P lyase-deficient mice [192]. However, in some animal models of brain ischemia, SphK2 activation is considered neuroprotective [207,208,209,210]. Whether SphK2 activation leads to neuroprotection or not may depend on the subcellular targets of S1P produced. The efficacy of S1P receptor agonists in neuroprotection in some of these studies [207,208,209] suggests that S1P produced by SphK2 may activate S1P receptors, leading to neuroprotection, or S1P in mitochondria [211] may exert cytoprotection as indicated in a myocardial injury model [212]. In contrast, S1P produced in other sites, such as nucleus and the ER, may have other targets and enhance apoptosis [191,192,194]. 

Our studies have demonstrated that ethanol exposure in P7 mice (5.0 g/kg, once) induces, within 2–4 h, transient activation of SphK2 and a similar transient increase in S1P in the brain. Because an inhibitor of SphKs, dimethylsphingosine, attenuates ethanol-induced apoptosis, S1P may enhance apoptosis in this system [180] (Figure 2). However, it has been shown that exogenous addition of S1P protects rat liver sinusoidal endothelial cells [213] and corneal epithelial cells [214] from ethanol-induced apoptosis. In 3T3 fibroblasts, ethanol enhances the stimulatory effects of S1P on both DNA synthesis and cell proliferation [215]. Our preliminary results [216] also indicate that SEW2871, an agonist for S1P receptor 1, which is a major receptor isoform in the brain [217] and is expressed mainly in astrocytes [180], attenuates ethanol-induced apoptosis in the P7 brain. S1P produced by SphK2 activation by ethanol may have a target different from S1P receptor 1, although our studies suggest that SphK2 is primarily localized in the plasma membrane/synaptic membrane of neurons in P7 mice, and not in the nucleus or the ER that are implicated as the sites for apoptotic action of S1P in the previous studies [191,192,194].

Recent studies have begun to uncover S1P functions in mitochondria. S1P reduces the membrane depolarization and the elevation in Ca^2+^ in mitochondria during oxygen-glucose deprivation [196]. S1P produced by SphK2 regulates complex IV assembly and respiration via interaction with mitochondrial prohibitin-2 [211], and the mitochondrial S1P is required for the downstream protective modulation of MPTP [212]. Although it is generally recognized that the balance between ceramide and S1P determines cell fate [7], mechanisms by which these sphingolipids reciprocally regulate apoptosis are not fully understood. Considering the roles of S1P and ceramide in mitochondria, elucidation of the coordinated action of these sphingolipids in mitochondria is important for understanding apoptotic pathways, including those triggered by ethanol. 

A recent study indicates that ethanol induces not only apoptosis but also autophagy in the P7 brain, and the enhanced autophagy formation is proposed to be the cells’ neuroprotective reaction [218], while ethanol suppresses autophagy in embryonic cerebral cortical progenitors, which are resistant to ethanol-induced apoptosis [219]. Both acute and chronic ethanol administration enhance autophagy formation in the liver [220,221,222,223,224] via ethanol oxidation [220,221,222,224] and through inactivation of Akt and the downstream mTOR signaling that controls autophagy formation [220,221]. Specifically, ethanol appears to enhance autophagy for removing damaged mitochondria and accumulated lipid droplets [221]. Recent findings indicate that sphingolipids are also involved in these autophagic processes (reviewed by [144,225,226]). The *de novo* synthesis of ceramide is reported to be essential for the induction of autophagy ([227,228,229], reviewed by [230]). Ceramide may induce autophagy through inactivation of Akt/mTOR signaling or up-regulation of beclin 1, which is required for the autophagosome formation (reviewed by [144,225,226]). It has been also reported that ceramide binds to LC3B-II and anchors LC3B-II-positive autophagolysosomes to mitochondrial membranes to induce mitophagy [231]. On the other hand, S1P has been reported to counteract amino acid deprivation-induced autophagy and cell death by suppressing mTOR inactivation through binding to S1P receptor 3 [232]. However, S1P produced by overexpression of SphK1 in MCF cells stimulates autophagy and attenuates apoptosis during nutrient starvation by increasing the formation of LC3 positive autophagosomes and the rate of proteolysis [233]. It is suggested that intrinsic regulation of autophagy by S1P is different from its extrinsic action via S1P receptors [232]. The S1P-induced autophagy is characterized by the inhibition of mTOR signaling without affecting Akt signaling and by the lack of robust accumulation of autophagy, and is considered cytoprotective [233]. Because the crosstalk between apoptosis and autophagy through sphingolipids (specifically ceramide and S1P) appears to be critical to determine cell fate [144,225,226], future studies of the possible involvement of sphingolipids in autophagy formation during ethanol-induced apoptosis are important to clarify the dynamic roles of sphingolipids in this apoptotic pathway.

## 5. Gangliosides in Ethanol-Induced Neuronal Apoptosis in the Developing Brain

### 5.1. Pro- and Anti-Apoptotic Effects of Gangliosides in the Brain

Gangliosides (sialic acid-containing glycosphingolipids) are particularly abundant in the nervous system and exert many biological functions as antigens, mediators of cell adhesion, and modulators of signal transduction (reviewed by [234,235,236]). Gangliosides are also known to be involved in apoptotic pathways. Specifically, GD3 ganglioside is reported to be pro-apoptotic in neurons [237,238,239,240,241], as indicated earlier in myeloid and lymphoid tumor cells ([242], reviewed by [243,244]). Also, elevated GD3 expression has been found in brain tissue with various neurodegenerative disorders ([245,246,247], reviewed by [248]). Although the majority of gangliosides are found in glycosphingolipid-enriched microdomains (lipid rafts) in the plasma membrane [235], GD3 accumulates within mitochondria of cells undergoing apoptosis [249,250], and direct interaction of GD3 with mitochondria induces cytochrome c release and caspase-3 activation [251]. There is also a report indicating nuclear localization of GD3 during apoptosis [238,252], which may affect histone H1 modification [252]. In addition to GD3, the involvement of GM3 in apoptotic death of dividing astrocyte precursors has been reported [253].

While some gangliosides mediate apoptosis in certain cell types, other gangliosides, specifically GM1, show anti-apoptotic/neuroprotective effects against cell damage caused by various types of stress and injury (reviewed by [21,22,23,24,25,26]). In serum-deprived PC-12 cells, exogenously added GM1 exerts anti-apoptotic effects through the augmented phosphorylation of NGF receptors [254], possibly by interacting with high-affinity Trk-type receptors for NGF [255]. GM1 increases phosphorylation of Trks (TrkA > TrkC > TrkB) and Erks in slices of striatum, hippocampus and frontal cortex of rat brain [256]. Thus, GM1 mimics or potentiates certain actions of neurotrophic factors (reviewed by [21,23]. It has been also reported that gangliosides activate Trk receptors by increasing the release of neurotrophins, such as neurotrophin-3 [257] and BDNF [258]. Activation of Trk receptors by GM1 leads to the stimulation of the PI3K/Akt pathway in the brain [259]. Although GM1 is mainly localized in lipid rafts in the plasma membrane, which contain many signaling molecules [235] including Trk receptors [260,261], it is also found in the nucleus. GM1 in the nucleus regulates nuclear and cellular calcium levels through sodium-calcium exchanger in the nuclear envelope and maintains neuronal viability [22]. Not only exogenously added gangliosides but also endogenous gangliosides increased by an enhancer of ganglioside biosynthesis confer neuroprotection in cortical neurons [262]. The neuroprotective function of endogenous GM1 has been also indicated in studies using animals or cells with totally or partially lacking GM1. Mice lacking B4galnt1 for GM2/GD2 synthase, which depletes GM2, GD2 and all the gangliotetraose-series gangliosides including GM1, are susceptible to kainate-induced seizures and neuronal apoptosis, and administration of membrane permeable derivative of GM1, LIGA20, attenuates the susceptibility of these mice [263]. Also, B4galnt1 knockout mice and their heterozygotes manifest Parkinson’s disease (PD)-like symptoms along with the loss of dopaminergic neurons, and such abnormalities are attenuated by administration of LIGA20 [264,265]. Also, decreased levels of GM1 found in cells from Huntington’s disease (HD) patients or the animal models contribute to HD cell susceptibility to apoptosis, which is restored by GM1 administration probably through Akt activation [266]. It has also been proposed that changes in the lipid rafts induced by ganglioside (including GM1) deficiency may cause neurodegeneration [24,236]. These studies indicate that endogenous GM1 in the lipid rafts and nucleus may contribute to neuronal cell survival.

The neuroprotective effects of GM1 thus observed provide foundation for clinical applications in neurological disorders, such as AD [267], Parkinson’s disease [268,269,270], spinal cord injury [271], and stroke [272]. 

### 5.2. Gangliosides in Ethanol-Induced Apoptosis in the Developing Brain

It has been shown that ethanol alters brain ganglioside metabolism. In adult rats, chronic administration of ethanol reduces GM1 and GD1a in the synaptosomal fraction and GD1a in the microsomal fraction with decreases in UDP-Gal: GlcCer galactosyltransferase and UDP-Gal: GM2 galactosyltransferase activities [273]. In addition, ratios of the long chain base C20 sphingosine/C18 sphingosine increase in GM1 of synaptosomes and microsomes and in GD1a of myelin [274], which may reduce membrane fluidity and affect the lipid-protein interactions in the lipid rafts [275]. Also, GD3 and GD1a decrease by augmented activity of sialidase [276], which appears to be the plasma membrane sialidase [277], and by reduction of CMP-NeuAc: GM3 alpha2,8-sialyltransferase gene expression [278]. Prenatal ethanol exposure on GD7 and GD8 and/or GD13 and GD14 increases GM1 and decreases polysialogangliosides in the fetal brain on GD20 [279]. However, chronic ethanol exposure during the gestation and lactation period increases ganglioside concentration in the offspring brain analyzed on P21 [280], while ethanol exposure during the gestation period does not significantly affect synaptic and axolemmal gangliosides in the offspring (P17 to P34) [281].

In cultured CGNs, ethanol increases sialidase activity and changes ganglioside profiles [48], and increases sphingosine recycling for ganglioside biosynthesis [50]. Importantly, ethanol exposure decreases the membrane GM1 content in the mouse neural crest cells, and a significant correlation has been found between the GM1 content and the viability of these cells [282].

In agreement with the general neuroprotective effects of GM1 described in the previous section, GM1 induces neuroprotection against ethanol toxicity. When added to culture media, GM1 diminishes ethanol-induced neural crest cell death and the membrane fluidity elevation [283], and provides protection against ethanol neurotoxicity in rat hippocampal neurons and in chick dorsal root ganglion neurons [284]. Our laboratory has shown that ethanol-induced apoptosis in rat CGNs is attenuated by pretreatment with GM1, GD1a, GD1b, GT1b, or LIGA20 [285]. LIGA20 was the most effective, followed by GD1b and GT1b, while asialo GM1 was ineffective. When administered *in vivo*, GM1 diminishes the teratogenic/toxic effects of prenatal ethanol exposure (reviewed by [286]). GM1 pretreatment blocks changes in ganglioside profiles, phospholipase A2 activation, and fatty acid ethyl ester production in the brain induced by fetal ethanol exposure in rodents, and minimizes the alteration in brain maturation and associated behavioral dysfunction [279,287,288]. Also, pre-administration of GM1 or LIGA20 attenuates ethanol-induced apoptosis in the P7 mouse brain [289]. These results indicate that gangliosides, specifically GM1, significantly attenuate brain abnormalities induced by prenatal or neonatal ethanol exposure, although roles of endogenous GM1 in the process of ethanol neurotoxicity in the brain have not been elucidated yet. 

In contrast, certain gangliosides, such as GD3, may function as an apoptotic inducer during ethanol-induced apoptosis. We have analyzed the time course of changes in ganglioside profiles in the brain of mouse pups exposed to ethanol (5.0 g/kg, s.c., one time injection) on P7 and found significant increases in GM2, but not in other major gangliosides including GD3. The small increase in GM2 observed 2 h after ethanol exposure is followed by a marked increase around 24 h [49]. GM2 may be associated with apoptosis, because GM2 is accumulated in cleaved caspase-3 positive neurons and increases in mitochondria in the P7 brain exposed to ethanol [49]. Furthermore, GM2, as well as GD3, induces cytochrome c release from mitochondria isolated from P7 brains. Interestingly, the addition of GM1 attenuates GM2-induced cytochrome c release from isolated mitochondria [49] indicating that the balance between GM1 and GM2 in mitochondria may affect membrane permeability. Thus, as illustrated in Figure 2, ethanol-induced apoptosis, which is promoted by ceramide/S1P, is attenuated by GM1 ganglioside. In addition, *in vitro* experiments suggest that GM2 may be pro-apoptotic. Although subcellular localization of these spingolipids remains to be clarified, it is tempting to speculate that the coordinate action of these sphingolipids in mitochondria may regulate ethanol-induced apoptosis in the developing brain. 

## 6. Conclusion

Ethanol induces acute apoptotic neurodegeneration in the rodent brain during the brain growth spurt period, corresponding to the third trimester of human fetuses. This apoptosis occurs via the Bax-dependent mitochondria-mediated pathway involving many factors, including ROS formation, inactivation of Akt and ERK, and activation of GSK3β. Although published studies are still scant, available data indicate that not only proteins but also sphingolipids are involved in the ethanol-induced apoptotic neurodegeneration in the developing brain. Ethanol alters sphingolipid metabolism profoundly in the developing brain as well as in cultured neurons. Particularly, elevation of *de novo* ceramide synthesis and S1P formation appear to mediate or enhance apoptosis. Ethanol-induced increase in GM2 ganglioside may also promote apoptosis, while exogenously added GM1ganglioside exerts the anti-apoptotic effect. Studies also indicate that ceramide and GM2 increase in mitochondria in the brain exposed to ethanol. The coordinated action of sphingolipids in mitochondria may be crucial for the regulation of this mitochondria-mediated apoptotic pathway. Some of these roles of sphingolipids, such as the pro-apoptotic action of ceramide and the neuroprotective action of GM1, are widely observed in neuronal apoptosis triggered by diverse apoptotic inducers. However, some of the effects of other sphingolipids may be specific to this ethanol-induced apoptosis, because of the unique and profound effects of ethanol on lipid metabolism in the developing brain. Obviously, further studies are needed to better understand the sphingolipid involvement in developmental ethanol neurotoxicity. These include; subcellular localization and trafficking of sphingolipids and related enzymes involved in ethanol-induced apoptosis, molecular species of ceramide and other sphingolipids altered by ethanol exposure, the relationship between glial activation and sphingolipids, the long-lasting effects of developmental ethanol exposure on sphingolipid metabolism, the involvement of sphingolipids in ethanol-induced autophagy, and the comparison of ethanol-induced apoptosis/autophagy between wild-type mice and the mice with disrupted genes for sphingolipid metabolism. These studies may help identify unique targets for therapeutic applications against FASD. 
